# Public health round-up

**DOI:** 10.2471/BLT.15.011215

**Published:** 2015-12-01

**WHO launches antibiotic resistance week**

The first World Antibiotic Awareness Week was launched last month by the World Health Organization to promote best practices among the general public, health workers, policy-makers and the agriculture sector and avoid the further emergence and spread of antibiotic resistance. The awareness week will run from 16 to 22 November every year.

http://www.who.int/mediacentre/events/2015/world-antibiotic-awareness-week

**Figure:**
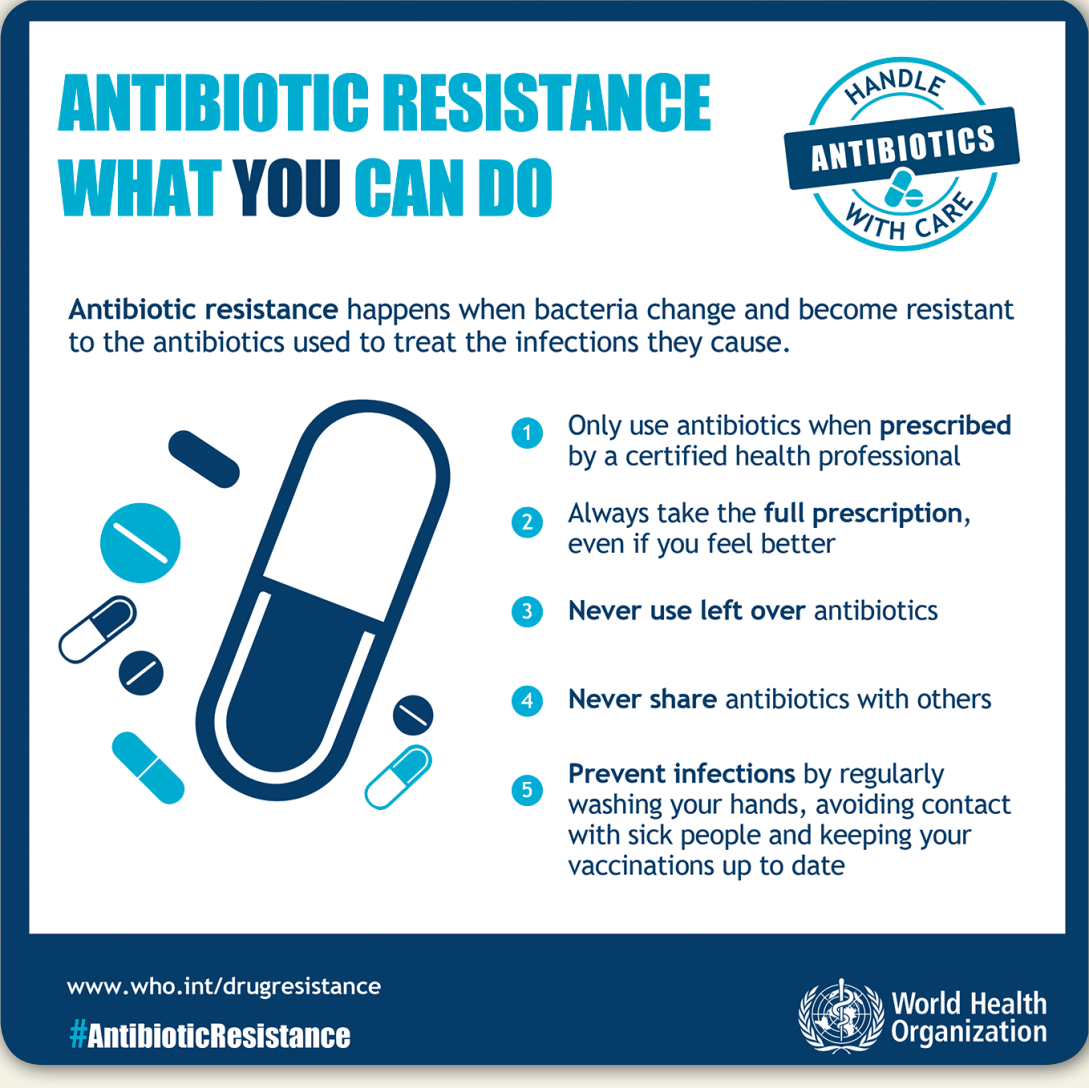
WHO

## Polio vaccine switch 

Each country and territory using the trivalent oral polio vaccine (that protects against three types of the virus) should switch to the bivalent oral polio vaccine (that protects against two types of the virus) during the last two weeks of April next year, a group of experts has concluded.

The Strategic Advisory Group of Experts on immunization (SAGE), which advises WHO, said that the 149 countries and seven territories using the oral polio vaccine should step up their efforts to prepare for the globally coordinated switch from trivalent oral polio vaccine to bivalent oral polio vaccine between 17 April and 1 May 2016.

Within those two weeks, countries should remove all stocks of trivalent oral polio vaccine and confirm their removal to WHO, the experts said.

Global withdrawal of the type 2 polio virus component in the oral polio vaccine is a crucial part of the polio endgame strategy: WHO’s plan to wipe out the disease.

It is the first phase of the planned removal of all oral polio vaccines, with the type 1 and type 3 components of the vaccines to be removed after the global eradication of all wild poliovirus types has been certified.

Withdrawing oral polio vaccine is crucial for eliminating very rare cases of vaccine associated paralytic polio (VAPP) or circulating vaccine derived polioviruses (cVDPVs). The type 2 component of oral polio vaccine accounts for 40% of VAPP cases, and more than 90% of cVDPV cases.

By contrast, wild poliovirus type 2 has not been detected anywhere since 1999 and, in September, the Global Commission for the Certification of Poliomyelitis Eradication declared this strain globally eradicated.

The SAGE cautioned, however, that more work needs to be done ahead of the April 2016 switch. 

A critical step is that the global community ensure poliovirus type 2 is appropriately contained so that it is not released into the environment, inadvertently or deliberately, to again cause paralysis or death in humans. 

 To do this, each country should implement the measures that are outlined in the WHO global action plan for containing polioviruses that was endorsed by the ministries of health of all 194 WHO Member States in May. 

http://www.polioeradication.org/

## Financing WHO

Representatives from Member States and other major financial contributors to the World Health Organization (WHO) gathered in Geneva last month to discuss how to ensure that the Organization’s programmes are fully funded in the coming 2016–2017 biennium. 

They joined WHO’s Director-General Margaret Chan and other senior WHO managers in reviewing the financing outlook for 2016–2017, as well as some key priority areas such as WHO’s role in contributing to the Sustainable Development Goals, accountability and WHO emergency reform. 

The Organization needs a total of US$ 4.4 billion – an increase compared with the 2014–2015 budget of US$ 3.977 billion – to accomplish the work planned for the next two-year period. 

Although WHO’s funding outlook for 2016-2017 seems positive overall, there are still a number of under-funded areas in 2016-2017 and the predictability for 2018-2019 is very low, according to the programme budget web portal. 

It was the second time that such a meeting, known as the financing dialogue, has been held to improve the transparency, alignment, flexibility and predictability of WHO’s funding. 

http://extranet.who.int/programmebudget/Biennium2016/Financing/http://extranet.who.int/programmebudget/Biennium2016/Financing

**Cover photo**

Farmers in Mongolia struggle with extremely cold winters, that they call the *dzud,* in which livestock die, as well as droughts in the summer. World leaders gather this month at the 2015 United Nations Climate Change Conference in Paris, France to reach a deal on reducing carbon emissions.

**Figure:**
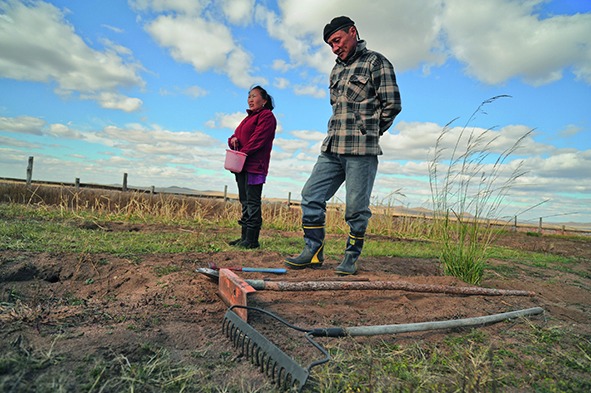
Asian Development Bank/Eric Sales

## Cholera in Iraq

A vaccine campaign was launched in Iraq last month to immunize some 250 000 displaced people and refugees across 13 of the country’s 19 governorates. 

The first round of vaccinations got under way in the first week of November. In early December, the second round of doses will be administered to complete vaccination in some 62 camps. 

The campaign was organized by the Ministry of Health with support from several international partners, including the World Health Organization (WHO), the United Nations Children’s Fund and the United Nations Refugee Agency. 

“The deteriorating security situation in Iraq coupled with the disruption of public health services and increased population displacement makes conditions favourable for transmitting the disease,” said Dr William Perea, coordinator of the Control of Epidemic Diseases Unit at WHO headquarters. 

WHO delivered the 510 000 doses needed, donated 600 000 chlorine tablets and 15 inter-agency diarrhoeal diseases kits, and sent epidemiologists and other experts to the country to support the campaign. 

As of 1 November, the Iraqi Ministry of Health reported 2173 laboratory confirmed cholera cases from 16 of 19 governorates since the start of the outbreak in September. Two deaths have been reported. 

More than 10 700 cholera cases and 170 deaths had been reported in the WHO Eastern Mediterranean and African Regions as of 21 October. 

To step up the response to prevent wider cholera outbreaks ahead of the rainy season, WHO needs more than US$ 5 million in funds. 

Cholera is an acute enteric infection caused by the ingestion of bacterium *Vibrio cholerae* present in water or food contaminated with faecal matter. Together with efforts to improve water quality, sanitation, and hygiene, vaccine has been proven to significantly reduce the spread of cholera in high-risk areas.

The overall coverage of the first round of the oral cholera vaccination in Iraq was 91% (227 000 immunized) of the target population, displaced Iraqis and Syrian refugees. This was first ever pre-emptive vaccination campaign with the oral cholera vaccine in camps for internally displaced people and refugees in Iraq.

http://www.emro.who.int/irq/iraq-news/who-mobilizes-510-000-doses-of-oral-cholera-vaccine.html

## Sierra Leone ends Ebola outbreak

WHO announced on 7 November that Ebola virus transmission has stopped in Sierra Leone. It said that 42 weeks – two incubation cycles of the Ebola virus – had passed since the last person confirmed to have Ebola virus disease tested negative for the second time.

The country has started a 90-day period of surveillance. If no cases are reporting during that time, the country can be declared Ebola free.

“Since Sierra Leone recorded the first Ebola case in May 2014, a total of 8704 people were infected and 3589 have died, 221 of them healthcare workers, all of whom we remember on this day,” said Dr Anders Nordström, WHO Representative in Sierra Leone.

http://www.afro.who.int/en/sierra-leone/press-materials/item/8139-who-commends-sierra-leone-for-stopping-ebola-virus-transmission.html

**SAGE: Request for nominations**

WHO is soliciting proposals for nominations for one current and several future vacancies on its Strategic Advisory Group of Experts (SAGE) on immunization.

SAGE is the principal advisory group to WHO for vaccines and immunization. Members should have an outstanding record of achievement in their own field and an understanding of the immunization issues covered by the group.

Nominations are solicited from all regions and should be submitted no later than 15 January 2016 following the instructions at: http://www.who.int/immunization/sage_nominations/en/index.html

**Looking ahead**

25–30 January – WHO Executive Board meeting in Geneva, Switzerland.

26 – 31 January – Prince Mahidol Award Conference, Bangkok, Thailand.

7 April – World Health Day. The 2016 theme is diabetes.

